# Strengthening collaboration within Dutch municipalities for a healthier living environment: experiences and possible improvements according to civil servants

**DOI:** 10.3389/fpubh.2024.1406178

**Published:** 2024-06-28

**Authors:** Kristine Mourits, Hilde Spitters, Koos van der Velden, Marleen Bekker, Gerard Molleman

**Affiliations:** ^1^Academic Collaborative Centre AMPHI, Department of Primary and Community Care, Radboud University Medical Center, Research Institute for Medical Innovation, Nijmegen, Netherlands; ^2^Tranzo School of Social and Behavioral Sciences, Tilburg University, Tilburg, Netherlands; ^3^Health and Society Group, Centre for Space, Place, and Society, Wageningen University and Research, Wageningen, Netherlands

**Keywords:** collaborative governance, collaboration, qualitative study, healthy living environment, boundary-spanner, public health, urban development

## Abstract

**Background:**

Health is partly determined by the physical environment in which people live. It is therefore crucial to consider health when designing the physical living space. This requires collaboration between the social and physical domains within municipalities. Collaboration is not self-evident, however, and it is difficult to achieve due to barriers relating to culture, language and work processes. Additionally, improvements in collaboration are desperately needed to address complex health issues, and working according to the new Environment and Planning Act in the Netherlands requires more collaboration. One relevant question concerns how civil servants describe the current collaboration between the social and physical domain and the concrete improvements they propose to improve such collaboration to build a healthier living environment.

**Methods:**

In this qualitative study, the Collaborative Governance framework was used to present data from semi-structured interviews with 21 civil servants in five Dutch municipalities. Respondents were asked to reflect on their current experiences with collaboration and suggest concrete opportunities for improving collaboration.

**Results:**

The results indicate that enhancing collaboration between the social and physical domains can be achieved by proceeding from the inhabitants’ perspective, as well as by encouraging aldermen and managerial personnel to take a more active and committed role in collaboration. This involves formulating and communicating a joint vision, in addition to guiding and facilitating collaboration through integrated assignments, forming multidisciplinary teams and appointing boundary-spanners. Civil servants see a clear role for themselves in the collaborative process. They recognize their own contributions to and obligations in enhancing collaboration by actively seeking contact, absorbing each other’s perspectives and pursuing common ground, starting today.

**Conclusion:**

There are many concrete opportunities to improve collaboration between the social and physical domains. This could be initiated immediately if civil servants, managers and aldermen approach collaboration as an essential part of their jobs and acknowledge the interdependency that exits.

## Introduction

The health of individuals is influenced by their biological condition and lifestyle, as well as by the availability of healthcare services and the social and physical surroundings (1, 2). A well-designed physical living environment can contribute to health in numerous ways. For example, the presence of good walking and cycling paths contribute to more exercise (3), and the presence of greenery contributes to mental health (4, 5) and social interaction (6). The efficient utilization of the physical environment thus has the potential to contribute to the ability to address a variety of health issues, including obesity, mental health issues and health disparities (7–9).

To capitalize on this opportunity, it is important to consider the protection and promotion of health during the development of spatial-planning projects (10, 11). This requires a multi-sector approach within municipalities, which involves collaboration amongst civil servants from diverse disciplines in both the social and physical domains (12, 13). The social domain concerns welfare, care, health, education and income, and the physical domain concerns housing, traffic, greenery and the design of public space. Such collaboration between domains brings together expertise and knowledge. On one hand, the social domain introduces topics relating to issues including the health needs of inhabitants and the influence of the environment on individual health. On the other hand, the physical domain contributes expertise on physical design and its possibilities, as well as on the ways in which other issues within the physical environment (e.g., housing, mobility and climate adaptation) can be combined with health aspects.

In the Netherlands, municipalities are responsible for the design of the physical living environment. Political governance is determined by the municipal council and carried out by the board of mayor and aldermen. In addition to the political administration, each municipality has an municipal organization responsible for the implementation of municipal policy. The municipal organizations are mostly divided into separate departments for social policy areas (the social domain) and physical policy areas (the physical domain). Collaboration between these domains is therefore limited, and the process of establishing it is not straightforward (14–16). Within such a compartmentalized municipal organization, it is more difficult for civil servants to find each other and to know what is going on in other policy areas (16). This makes it difficult to generate attention to health in spatial-planning projects. In addition, as demonstrated in several studies, factors other than organizational structure also contribute to the perception that collaboration between the social and physical domains is challenging (17, 18).

The social and physical domains differ in terms of language and culture (19), as well as with regard to working processes (20). Projects in the physical domain involve concrete, visible projects made of stone and greenery, with a strong business case and clarity about costs, focusing on the long term and yielding clearly visible results. In contrast, the social domain operates through a process of collaboration between people, making agreements and implementation. In this domain, results are often not immediately apparent and, in many cases, they cannot be directly attributed to any specific action.

The challenges associated with collaboration between the social and physical domains are even more prominent for the concept of health. For example, the broad nature and definition of health (21) makes it challenging to pinpoint the elements that should be incorporated into spatial-planning projects. Furthermore, the effect of a physical measure on the health of inhabitants is visible only in the longer term (22). Furthermore, because of the multi-layered determinants of health, it is not possible to establish the direct impact of a given measure on the health of inhabitants (1, 2, 22).

Despite the perceived challenges, collaboration between the social and physical domains is crucial. This is because the only way to address complex health issues is through an integrated approach with actions in diverse disciplines other than health (23). Collaboration is seen as a crucial tool for resolving complex social problems, and it has therefore been investigated by numerous scientific disciplines, including public administration and public health. The literature uses a variety of terms to refer to collaboration (e.g., intersectoral action, interdisciplinarity, cross-sectoral collaboration), and it presents various models of collaboration (24, 25). At the same time, however, the literature lacks a clear, widely accepted definition of collaboration (26). In an examination of consensus concerning the composition of collaboration, Mayer and Kenter identify nine elements: communication, consensus in decision-making, diverse stakeholders, goals, leadership, shared resources, shared vision, social capital and trust (26). In addition to these elements, D’Amour emphasises that bringing disciplines together does not directly lead to collaboration. Organizations should have a structure in place to facilitate the process of collaboration (27).

In this study, we examine developments in collaboration between the social and physical domains in Dutch municipalities. Within this context, it is becoming increasingly accepted that collaboration between domains is essential in order to address health issues. Many municipalities are actively working on this (13, 28–32), although it remains challenging. In the Netherlands, developments in the areas of health, climate and inequality, combined with the introduction of the new Environment and Planning Act have rendered the obligation to shape such collaboration properly and structurally stronger than ever before.

Prior to the implementation of the new Environment and Planning Act in 2024, we conducted a study of Dutch municipalities based on the following research question: *How do civil servants describe the current collaboration between the social and physical domains, and which concrete improvements do they propose to improve such collaboration in order to build a healthier living environment?* The findings suggest concrete recommendations for Dutch municipalities concerning important elements for strengthening collaboration, as a crucial factor in establishing a healthier living environment and resolving intricate health concerns.

### Theory

The process of gathering data for this study was guided by everyday practise of research K.M., the research question and the well-documented barriers that have been outlined in the literature. We compared our analysis and interpretation of the data to existing scientific models. Different theories such as Sectoral Collaboration (Bryson) (33), Intersectoral Action (Mondal) (34), Collaborative Governance model (Ansell and Gash) (35) and Integrative Framework for Collaborative Governance (Emmerson) (36), have been viewed. The Collaborative Governance model works well for collaborations between individuals and departments of organizations who are in the early stages. Also, this model is based on a meta-analytical study of existing literature and 30 empirical case studies (35), which provides opportunities for learning within the field of public health. Collaborative governance refers to an explicit, formal strategy of incorporating stakeholders into multilateral and consensus-oriented decision-making processes. The four components of the collaborative governance model are the collaborative process, facilitative leadership, institutional design and starting conditions (35). Collaborative-process variables form the core of the model, which is presented as a cycle, given the highly iterative and nonlinear character of the process. The other components (i.e., attention to trust, power, resources and knowledge on the start of the collaboration process, institutional rules and available leadership) are important contributors to a supportive context within which the collaborative process takes place (35). Good collaboration within the municipality is important to the ability to communicate jointly and unambiguously with external stakeholders and inhabitants. Our study therefore starts by concentrating on collaboration within one municipality before examining collaboration between municipalities and other stakeholders.

## Materials and methods

### Design

**Figure 1:**
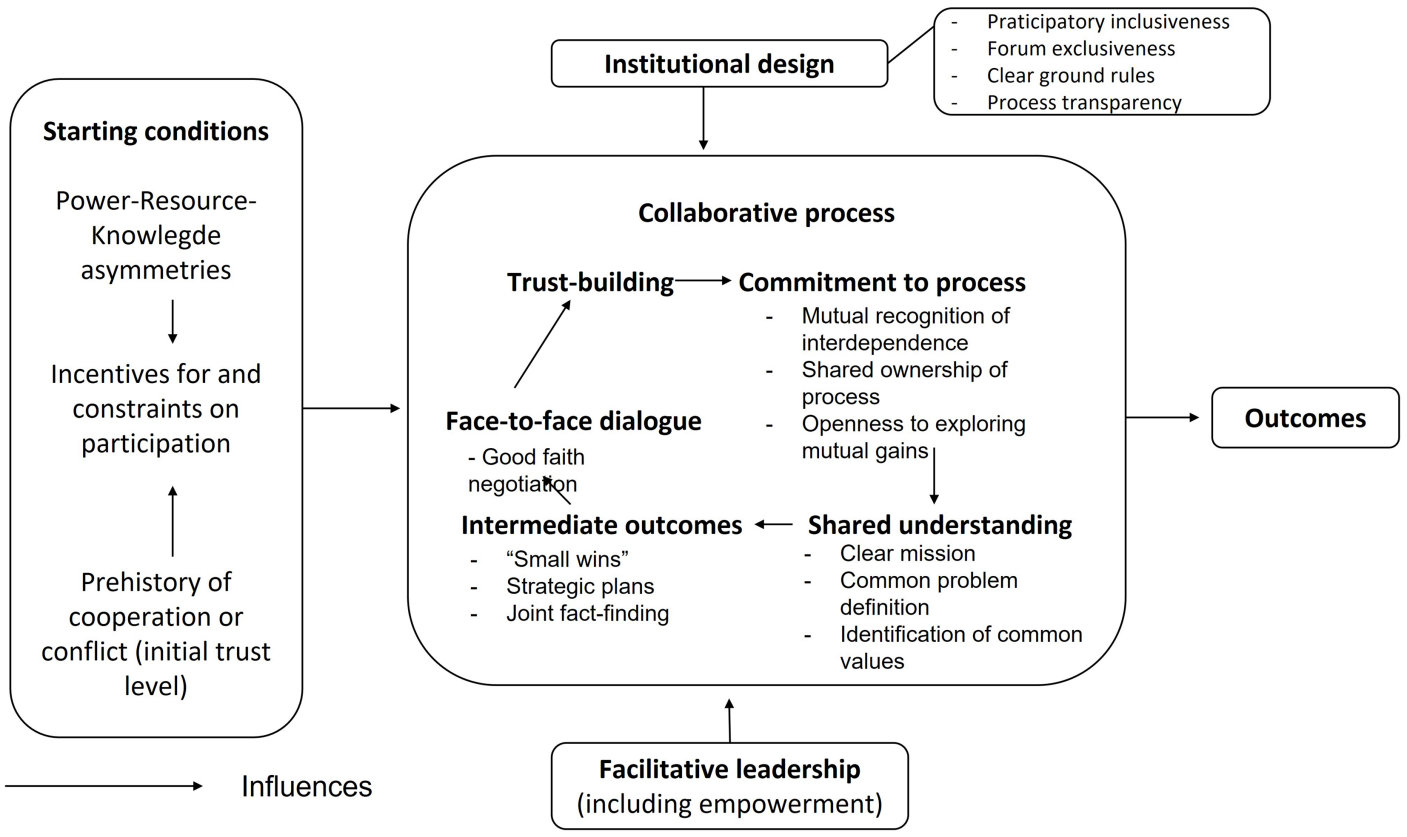
Model of collaborative governance. Reprinted with permission from Oxford University Press from Ansell and Gash (35), Copyright © 2007 the Authors of (35); published by Oxford University Press on behalf of the Journal of Public Administration Research and Theory, Inc.

In this qualitative study, we sought to identify similarities amongst municipalities regarding their experiences in collaboration across domains, along with potential perceived improvements. Semi-structured interviews were conducted with civil servants from different policy areas in five Dutch municipalities. This is an effective data-collection method for a detailed exploration of the views and experiences participants (37). The study was carried out in a collaboration between two consortia (Space2move and GELIJK) within the ‘Maak ruimte voor gezondheid 2018–2022’ (Make space for health) programme (38) operated by the Dutch funding organization ZonMw. This programme consisted of seven regional consortia of practitioners, policymakers and scientists each conducting a study on the effects of environmental planning on health, sustainable (un)healthy behavior, and participation in society. A part of the research conducted within this programme also focused on strengthening collaboration and implementation of a healthy living environments. The medical ethics committee for the Arnhem-Nijmegen area (2018–4252) and the ethics committee of Tilburg University (number RP211) approved this study, which was conducted according to the principles of the Declaration of Helsinki (October 2013, 64th WMA General Assembly) and in accordance with the Dutch Personal Data Protection Act. Written informed consent was obtained from all participants prior to data collection (Figure 1).

### Recruitment of the municipalities and participants

The selection of the five municipalities was with purposive sampling in a different cases design based on the involvement that the municipalities had with healthy living environments. The most important factor for inclusion was the municipality’s participation in the ZonMw ‘Make space for health’ programme (38). In addition, we asked municipalities that were involved in the Gelderland City Network, which focuses on collaboration and sustainability in relation to health and well-being. Five of the ten available municipalities ultimately decided to participate in this study.

The municipalities included in the study varied in terms of a wide range of demographic characteristics (e.g., number of inhabitants, urbanization, number of employees), and the participants differed according to position, gender and years of experience (Table 1).

**Table 1:** Population and participant characteristics of the municipalities included in the study.

**Municipality**	A	B	C	D	E	Netherlands
Total number of inhabitants^1^	161,348	117,165	232,874	92,423	17.429	17,407,585
Degree of urbanization^2^	1,649	368	1,255	1,738	780	517
Number of municipal employees^3^	Approx. 1,000	Approx. 850	Approx. 3,000	Approx. 800	Approx. 130	Approx. 160,000 municipal civil servants
						**Total**
Study						
Number of participants	4	4	5	5	3	**21**
Position Civil servant, social domain, public health Civil servant, physical domain, built environment Civil servant, focus on the Environment and Planning Act	1 3 0	2 2 0	1 2 2	1 2 2	1 2 0	**6** **11** **4**
Gender Female Male	0 4	2 2	3 2	5 0	2 1	**12** **9**
Years in position ≤ 1 1–5 5 or more	1 1 2	1 0 3	0 2 3	3 1 1	0 1 2	**5** **5** **11**

^1^Characteristics as of 1 January 2020 from Statline (Statistics Netherlands).

^2^Degree of urbanization (number of inhabitants per km^2^).

^3^From the municipal budget or social annual report.

Given our focus on collaboration between domains within municipalities, for the selection of the participants, we sought to incorporate a variety of civil servants from each participating municipality. More specifically, we selected a civil servant with a focus on health (social domain), one with a focus on the built environment (physical domain) and one who was tasked with implementing the Environment and Planning Act. The selection of interviewees was strategic. We started by reaching out to a point of contact in each municipality. This person was asked to personally approach three or four colleagues to participate. This resulted in a total of 21 participants, varying from three to five civil servants per municipality.

### Procedure

The data were collected between November 2019 and March 2020. The topic of collaboration between the social and physical domain in the Netherlands gains renewed interest and relevance with the introduction of the Environment and Planning Act in 2024. Some of the results in this article were confirmed in a more recent study (data collection sept- nov 2022), while other findings in this paper have not been countered in our later study data. Most of the interviews were held in person, but some had to be held through video-calling, due to the COVID-19 pandemic. The interviews were audio-recorded and transcribed verbatim. The duration of the interviews ranged from 60 to 90 min.

In addition to generating the data for this study, the interview guide used (Supplementary Appendix Table A1) led to an article on the spatial-planning process and the focus on health during this process (submitted). The interview consisted of the following parts: job description, integrated assessment processes, collaboration between the social and physical domains, health in spatial-planning projects, the Environment and Planning Act, and general information. The interview guide also included two simple instruments. The first instrument asked respondents to rank (in order from most to least experience) a list of obstacles known from the literature (17–20): different problem definitions, conflicting interests, differences in language and culture, lack of mutual understanding, and differences in short-term and long-term vision. The second instrument was used to identify the extent to which the municipalities paid attention to health in spatial developments. We used the maturity model developed by Storm and colleagues, which consists of five stages: unrecognized, recognized, considered, implemented, integrated and institutionalized (39).

The main investigators of this study, the authors K. M. and H. S, recruited the participants and conducted the interviews. In addition, a third interviewer was employed by Radboud university medical center. This interviewer received instructions from K. M. with regard to data-collection and interview techniques.

### Analysis

The data were analyzed using the AtlasTi 8.1 software package. Transcripts were analyzed according to open coding (40, 41), starting from the main aspects of this study (e.g., current collaboration, definition of domains, enablers and challenging elements, personal attitude towards collaboration, personal experience with collaboration, aspects of institutional design, and ideas for improvement in collaboration). The ranking of obstacles to municipal collaboration (instrument 1) and the level of attention paid to health in spatial development (instrument 2) proved to be highly interrelated and have therefore been analyzed and processed in an integrated way. To establish inter-rater reliability, the authors K. M. and H. S. coded the first two transcripts separately, after which they compared and discussed the codes until consensus was reached. This process resulted in the codebook that was used by one author to code all other transcripts, and by the other author to checked these codes. In this step as well, differences were discussed until consensus was reached. In all, 27 codes of the 65 codes were used for this article. The other codes were used for another article (submitted). In the next step of the analysis, the data were examined separately for each domain, before the sets were put together.

## Results

This section begins with a broad description of the ways participants experienced the current collaboration between domains. We then use the four components of the model developed by Ansell and Gash to present the results concerning possible improvements for collaboration. Presented in Box 1 are the improvements that participants from the social and physical domains mentioned for each of the domains.

### Current collaboration

The new Environment and Planning Act encourages civil servants to work together in both the social and physical domains. Nevertheless, concrete spatial-planning projects are regarded as the most important reason for collaboration. Such projects bring together civil servants from different policy areas, as coordination amongst these areas is required with regard to what needs to be achieved (e.g., specifications for layout, minimum area requirements or the possibility of combining diverse functions).

In recent years, all participants from all municipalities addressed in this study had experienced an increase in contacting each other, finding each other and initiating collaboration. At the same time, however, collaboration continues to occur on an *ad hoc* basis. It is highly dependent on how individual civil servants interpret their organizational task descriptions if the collaboration occurs. In most cases, it takes place within the specific domains of the individual civil servants. The participants noted that their experiences have been positive when seeking collaboration simply by reaching out to and starting to communicate with other parties. During this initial step, they get to know each other better and gain a higher level of comprehension, thereby enhancing their understanding of each other and their respective working methods. The aspects of communication emerging in this step (e.g., proactive thinking, talking with each other, transparency) and cross-pollination (as a means of building relationships and social capital) thus apparently provide a good foundation for collaboration.

According to the participants, collaboration depends in large part on the project or assignment. When the project description encourages collaboration between domains, other disciplines are often more actively and consciously involved, and overlapping themes are explored. One aspect that encourages collaboration is the designation of a project leader, who is charged with seeking common ground across disciplines. Another factor that participants mentioned as having a positive impact on their working attitudes towards collaboration occurs when aldermen advocate and promote structural collaboration across disciplines. Collaboration is facilitated by some specific themes (e.g., sports or local environment vision documents) in which common ground between the social and physical domains is clearly visible. Collaboration between domains is also encouraged by bottom-up approaches proceeding from the perspective and needs of neighborhood inhabitants.

As noted by the participants, the collaborating activities described above require hard work. One reason is that, in many cases, the social and physical domain continue to be perceived as two separate worlds, each with its own working attitudes, structures, knowledge base and task descriptions. The social domain is oriented towards people and the short-term enhancement of liveability, whereas the physical domain focuses on the built environment and the long-term enhancement of liveability. Time and practice is needed to overcome this perspective, together with a feeling of intrinsic motivation to take steps in favour of collaboration.

So, that’s starting to come together a bit now. I think the environmental teams have a very important connecting role in this regard, because they literally do bring things together. So they also see how compartmentalized our work still is, even though we think we’re working in a highly integrated manner. And so, I do indeed think that, with the Environment Vision, we’ll also make those connections. So that connection can now be seen from both sides. (Quotation 1, Municipality A)

### Elements that influence collaboration and concrete steps for improvement

The participants mentioned several elements that influence collaboration, both positively and negatively. Although they are highly entangled, we used the four components of the Collaborative Governance Model developed by Ansell and Gash (Table 2, Columns 1 and 2) to structure these elements in order to enhance interpretation. Given our primary objective of identifying necessary improvements, the following text focuses largely on this issue, as presented in Column 3 of Table 2.

**Table 2:** Elements of collaboration, current status and concrete steps toward improvement.

	**Current status**	**Concrete steps toward improvement**
**Starting conditions**		
Complexity (of health issues)	Complexity is an overall theme in many topics and issues (e.g., addressing obesity, preventive measures, psychological problems relating to the spatial area). The difficulty and complexity of the issues can increase the likelihood that they will remain unaddressed. This raises the question of ‘where to begin’. An assignment can be challenging enough in itself, let alone when it involves other disciplines or a combination of assignments.	Politics and management should ask for or demand an integrated approach, thereby resulting in a process that starts together in overall programmes and departments (see also Institutional Design: Thinking in silos). Employ people who are able to look across disciplines and consider the overall picture, in addition to the experts who know their own topics (see also Institutional Design). Achieve results with small projects (quick gains) and continuing speed, whilst also working together on larger, more complex projects.
Vision	The overarching vision of the municipality is unknown. Relevant questions include: What is the ambition? What does the municipality stand for? How this is translated to the other levels in the organization? This vision is especially important, as it is easy to become carried away by day-to-day issues and maintain a focus only on the assignments of one’s own department. Differences between the social and physical perspectives can be observed in the short-term and long-term vision. The perspective of inhabitants is often not addressed sufficiently.	Look beyond the tenure and vision of the alderman. Politics should have and communicate a coordinated, overarching vision, which the management can translate into joint assignments. To keep them feasible, these assignments can be divided into subtasks. One helpful way to translate a vision in actions oriented towards a better living environment is to devote sufficient attention to the perspectives of inhabitants in visions and taking the inhabitants as a starting point. Future costs and benefits should be considered in both the short-term and long-term vision.
Prehistory of collaboration	A system of ‘us-versus-them’ thinking is not helpful. Lack of comprehension can arise due to former organizational structures, hierarchies and generation gaps. There appear to be prejudices towards the other domain. Openness to other perspectives is experienced as a barrier. There appears to be a narrow view of task descriptions. Collaboration takes place only occasionally.	For civil servants in different domains to be on speaking terms, they must first meet each other and engage in conversation, thereby getting to know each other (see also Institutional Design: Thinking in silos). Start with a conversation, in which the focus is on questions and listening and not on giving the answers yourself. Attend project sessions on a regular basis, and sit at the same table for a similar neighborhood. This stimulates both the connection between disciplines and working together in projects. Approach collaboration as part of the job.
Lack of the full picture	Processes are not fully in view. This concerns both what it takes to work towards an integral process and what a spatial planning process looks like. It seems like an overwhelming process. Those involved lack a sufficient overview of what is going on in their own or the other domain, and they therefore missing the linkages necessary to establish common ground. A good overview is also missing due to the lack of integrated policy documents and the impossibility of reading and knowing all documents of the other domain. Those involved do not know with which stakeholders the other domain is collaborating. Lack of the full picture has a function: It maintains a focus on the tasks and aims of a particular domain, thereby allowing the processes within that domain to continue without becoming more complex. “That’s the way it is. You cannot know everything.”	Acknowledging each other’s processes, listening to each other’s contribution and really hearing each other can stimulate the collaborative process. Create an organizational chart to enhance visibility. Visualize the documents, so that it becomes clear where connecting are between policy areas. Initiate a conversation and ask clarifying questions to yourself and the other person when exploring the collaboration: Do you need to have an idea of what the other person is doing? Do you need insight into the other person’s work?
Resources	Lack of room to make adjustments in budgets and resources. External resources have the disadvantage that those who receive the resources might not have the necessary knowledge and expertise (regarding the needs of the target group).	There is a desire for flexible budgets or the use of external resource opportunities. Look for resources and incentives outside the municipality (e.g., from the national government) to create staff time for exploring connections and common ground. These resources are intended to stimulate collaboration.
**Collaborative process**		
Mutual understanding Common ground (commitment to process) (shared understanding)	Understanding of each other’s problems has increased over time, and it is created by investing in: Mutual respect Realising that all those involved have and must cope with their own work dilemmas and needs Having the conversation with each other and not proceeding purely according to documents Knowing what the other domain looks like Such investments help to concretize and acknowledge differences in opinions while continuing the conversations on a regular basis. The benefits of collaboration in terms of surprising new ideas and solutions are not always recognized. Some people experience discomfort on the way towards collaboration, as they lack a clear idea of how to collaborate. The search for common ground can be accompanied by greater complexity and a feeling of being overwhelmed. This could lead individuals to stop their own actions and project their experiences onto others, thus generating a negative snowball effect (see also Starting Conditions: Complexity).	The collaborative process should proceed from the acknowledgement that all the work is done for the benefit of the inhabitants. This immediately provides the most important common ground crossing all domains. Civil servants need to understand it as such: they all have the same ambition. Taking the neighborhood, the target group and/or the municipality as a starting point can help to reveal common ground and concretize what and who is needed from both the social and physical domain. Collaboration arises in the active search for common ground in the vision, project or assignment. Be attentive to which disciplines should be involved. One initial step in this regard could be to look at your own objectives and see how they fit into another theme. This makes it possible to move forward together by using in-depth sessions. It can also create alignment and sustain mutual understanding, thereby investing in expectation management. Another option could be to work with mutual assignments, with each group targeting them from their own perspectives and seeking common ground. Be aware of points at which objectives interfere. This is just as important as common ground. Health is regarded as a theme with clear overlapping aspects for both the social and the physical domain.
Time	The lack of sufficient time to invest in the other domain is experienced as a barrier. Collaboration takes time, both within and between domains. A different way of working requires time investment (e.g., starting with assessment management or using a bottom-up approach by getting to know the needs of the target group or neighborhood).	Acknowledge that change takes time. Make room to understand each other, and give the collaboration time to grow.
Work pressure	The feeling of pressure creates the impression that there is not enough time or space to consider the broader context, to look over borders or to accept invitations. Work pressure leads people to continue doing what they have always done in order to complete their tasks, as this is what they are expected to do. Work pressure increases when people are expected to consider all interests. In turn, this increases tension on the integral approach. This is impotence, not unwillingness. Work pressure increases due to a lack of capacity. Work pressure increases when people perceive a lack of standard routines and the need to work everything out for themselves. It is assumed that the other person is busy. Work pressure diminishes creativity.	Approach networking and achieving alignment as part of the job; incorporate it into the job description. Dare to set aside the feeling of work pressure, and use breaks to have conversations with colleagues. If you receive an invitation but do not have time to attend, ask a colleague to attend instead. Create a new position with the assignment to take over tasks from colleagues to reduce work pressure and retain progress.
Problem definition	Differences in problem definition develop when only the perspective of one domain, programme or discipline is considered. In many cases, the experience is that people tend to place the interest of their own domain above that of the other, partly because one definition of a problem is missing. Domains look at issues from different perspectives: the physical domain is concerned with the larger picture, whereas the social domain considers the needs of individual people. Those who do not experience problem definition as a barrier tend to think that they have a quick general understanding of the problem, which they perceive as sufficient. Participants wonder to what extent the physical domain health see as a problem.	Acknowledge that different people have different points of view, and they are therefore likely to have different definitions of the problem. Starting from the same base helps in the definition of the problem. This is possible when there is a mutual assignment. The inhabitants should also be considered and heard in this approach.
Conflicts of interest (intermediate outcomes)	Conflicts of interest arise when problems are approached differently or from different starting points, visions or goals. The ways in which budgets are currently allocated make it difficult to overcome conflicts of interest. For the physical domain, conflicts of interest occur primarily within that domain, and not between domains. Focusing on the target group is apparently a barrier to short-term solutions, as long-term plans are needed in order to reorganize a neighbourhood. Differences exist between organizational interests (e.g., we want similar things for the inhabitants) and personal interests (e.g., my own thoughts and objectives towards a subject).	Engage in conversation to reveal the conflicts of interest, to acknowledge their existence and to search for solutions. When conflicts of interests are revealed, one solution could be to engage a higher level in the hierarchical organization. Engage regular dialog about the health-related preventive measures that have been implemented in spatial planning. What does this entail for the long term?
Organizational drawbacks to collaboration Changes in the institutional design	Organizational changes from within are needed, in terms of both atmosphere and mindset. Investment in time or lowering the threshold to sitting together, seeking each other and taking the first step. This needs to be organized. Reorganization will not immediately provide the feeling of always having time for collaboration. More is needed. In general, it is still not common for projects to stimulate collaboration. Informal structures seem less common in larger organizations or when people are not in the same programme and are not direct colleagues. There is a lack of incentives. The administrative process is seen as a bottleneck. It is also inefficient to have too many colleagues at the table.	A process facilitator is needed in terms of both content and integrated process. One practical example is having a communication system to facilitate collaboration within the municipality. The foundation of the institutional design could be to aim for integrated assignments throughout the entire organization, from aldermen to executive staff. These assignments should be paired with the necessary financial resources. Address questions concerning various aspects, including which internal processes should be in place and how dialog can be organized at and between levels. Acknowledge the impact of meeting corners and allies. This is where the first contacts arise, for small talk and knowing who is around. Making the physical distance smaller can help you literally to cross paths, which in turn facilitates the first contact. Connections and starting to network can be stimulated by having everyone in the same building, as this makes it easier to meet each other. At the same time, it remains important to ‘just approach each other’.
Thinking and working in silos	Thinking in silos is also seen at national level, and this is transferred to the local level. Tasks are strictly divided in the organizational structure, and this does not enhance collaboration between domains. Each policy area wants to claim its own topic and core business by developing a vision and incorporate it into a policy document, without considering other disciplines and domains. This attitude stimulates thinking in silos. In addition, an integral approach is often started from the perspective and objectives of one’s own domain before other domains are considered. When connections seem to have a negative impact on personal objectives, possibilities for collaboration are neglected. This works both ways. Asking for attention to one’s own topics is likely to be neglected by others. People are held accountable for their own interests and objectives. These interests weigh more heavily than the common interest, thus maintaining the tendency to think in silos. Work pressure has a negative impact on thinking in silos. It maintains the focus on one’s own tasks. Thinking in silos persists because of how it is organized: with separate islands and projects, thereby excluding tight connections and structural embedding. A direct relationship appears to exist between thinking in silos and budget allocation, physical distance, bad communication, and the timing of informing the other party. The positive side of thinking in silos is the clear division of expertise and knowledge. The environmental theme in particular is perceived as keeping silos intact, given that strict legal regulations leave only a small margin for negotiation. For example, with regard to housing, environmental themes are considered, but not from the broader health perspective of the social domain.	It is recommended to change the organizational framework to overcome the tendency to think in silos. Write policy documents together, considering each other’s domains and working towards integrated assignments into which accountability is incorporated. Take the inhabitants as a starting point to overcome silos, when considering specific themes (e.g., healthy living environment and quality of urban life) as part of environmental law (see also Vision and Common ground). At the personal level, experiencing the feeling of time appears to be an important factor in taking the time to look across silos. A learning aspect is the existing wish to learn to adopt a more generic perspective and to merge themes instead of accumulating aspects. One practical example could be to execute impact tests as a purposive way of addressing the positive and negative impact of separate programmes. This is insightful and resourceful for aldermen, as well as for facilitators and programme leaders.
Asymmetries of culture and language	Differences in culture and language are regarded as an overarching factor that influences all other factors. Culture differences are interwoven within the organizational structure. This depends on aspects including assignment, role in the organization, type of work, us-versus-them culture, type of people in the domain and gender. One common thought is that “Social does not deliver, and Physical does not listen.” Different disciplines use different words and definitions for similar issues. The social domain is fuzzy, whereas the physical domain is right to the point. Most civil servants consider issues from their own perspective. This is partly because each domain has its own background and expertise. Health is not a standard theme at the table, and no standard definition is used.	Dare to dig deeper and find the root cause and meaning of what someone says until you truly understand what has been said. Acknowledge that perspectives differ from person to person and from role to role. Dare to take a different perspective and starting point. Acknowledging cultural and language differences across domains can facilitate change. The use of similar definitions can help people in different domains to speak a similar language.
	At the political/management level, personal interests and ideas seem to play a greater role. At present, collaboration is still highly dependent on individual initiatives and the informal structures within the network.	Cultural change and overcoming silos can be initiated by developing a shared vision for the council of aldermen. Assign the leaders of overarching programmes to facilitate connections between people. If aldermen spread the vision and convert it into programmes, the programme staff could take over the implementation and execution of these programmes. In their turn, leaders should work together to identify common ground between programmes, as well as at the executive level. At the policy level, create mutual understanding by investigating similarities and differences across domains, resulting in knowing when to compromise.

### Starting conditions

Complexity emerged in connection with many issues and topics that the participants reported addressing in their work. Although complexity in itself provides common ground between domains, it can also quickly become overwhelming, thereby generating even more complexity. To overcome this, it could be helpful for the municipal organization to have starting conditions that facilitate collaboration. For example, to overcome the complexity of social and health-related issues, the municipal council (consisting of the mayor and aldermen) and managers could demand integral approaches that lead to overarching programmes. In addition, a clear municipal vision, clearly expressed by all aldermen, resulting in joint assignments would have a positive influence. The participants further noted that these starting conditions subsequently provide incentives for collaboration, as they create a sense of empowerment by the organization. The incorporation of collaborative activities into job descriptions was also perceived as helping civil servants to focus on the overall vision of the municipality, instead of on their own tasks.

We’re taking health as a starting point, quality of life. We’re really going to do it all differently. It’s all there in the council’s programme. That was another huge boost. At least for the next four years. This council’s just going full steam ahead with this. They’ve even added a major plus to it. It all helps. Then everyone also knows that … so, now you really don’t have to explain any longer. (Quotation 2, Municipality C)

In the opinion of the participants, joint assignments could also help to address resource imbalances, as budgets currently tend to be organized from the top down, through separate programmes and departments, instead of horizontally, through overarching programmes or from the bottom up, in alignment with needs of the inhabitants. The neighborhood approach was mentioned as a helpful starting point, in which budgets are easier to merge or transfer.

Something else that was also very important in this process was the whole move towards area-based working or district-based working. Everything we do in the municipality now, we do from a district-based perspective. … really organize some things at district or area level. So, that means that we have to start actively working with the people from the physical and social… we really have to build bridges between physical and social. And so, that’s really happening in the district, the way we’re working together. (Quotation 3, Municipality C)

In addition to the integrated organization of budgets, participants placed high value on bottom-up approaches proceeding from the needs of inhabitants within the community, as well as on area-focused projects, as they provide a similar starting point for both the social and the physical domain. This brings both domains together at the table directly from the beginning, resulting in a smooth stimulus for collaboration. It is also helpful to build on a positive history of collaboration, overcoming several gaps (as mentioned in Table 2, under ‘Prehistory of collaboration’).

Another element of starting conditions that participants mentioned was ‘lack of having the full picture’. This occurs when civil servants do not have sufficient knowledge of what is going on in the other domain in terms of projects and working processes. Although participants noted that this element could also be functional (by maintaining a focus on individual tasks and aims, instead of adding complexity), they also acknowledged their desire to have a more clear picture of what was going on in their organizations. Ideas for how this can be done included making use of visualizations (see Table 2).

A colleague of mine is working on playgrounds in every neighbourhood, and I have an assignment from the national prevention agreement to make playgrounds and play facilities smoke-free. So I’ll go there right away, because it's obviously not on my colleague’s radar that we’ve been given that objective. But then, of course, we first need to know which of us is working on what… (Quotation 4, Municipality D)

According to the participants, resources at the provincial or national level can be used to support collaboration when seeking a broader perspective on how to improve a healthy environment. This can be accomplished through programmes aimed at collaboration, as well as through funds intended to stimulate collaboration. One disadvantage of this element has to do with the fixed amount of budgets, which neglect the amount of time that the collaborative process can take before the actual collaboration can start.

### Collaborative process

Every participant expressed willingness to collaborate and understand their own responsibility. During the interviews, participants were aware that collaboration is accompanied by individual effort in personal contact, thereby acknowledging their own influence on the process. They also reflected directly on their own shortcomings with regard to taking action in this regard. According to the participants, the collaborative process starts with having an open attitude towards each other’s expertise and working methods; genuinely listening to each other in order to truly understand what the other needs; ‘being able to translate needs to actions’, knowing where to start and proceeding from the assumption that others are available and willing to collaborate. Mastering these specific skills could be beneficial for the collaborative process, as it builds on mutual understanding and creates a good base for finding common ground.

During the interviews, participants acknowledged that short lines of communication are essential to remaining informed. One way to ensure this could be to build further on existing contact and to establish and deepen the connection, given the ongoing processes that collaboration entails. Another way could be to ‘simply’ initiate the contact, to get to know each other, to learn the work that each other does and to see common ground between disciplines. After the first contact has been made, especially when people see that someone is interested in their work, they are more likely to involve them in future projects. This builds on intrinsic motivation. Finally, participants mentioned that the organization of orientation meetings could be beneficial as well, or establishing connections with colleagues through the use of online communities (e.g., WhatsApp groups or the intranet).

… It’s also incredibly funny to see that it’s also a lot of fun when we let people have a say at an in-depth session like that, and that they also think, “Gee, it really is fun to be here at the table together. To do this together.” So, it’s all practice, practice, practice. (Quotation 5, Municipality C)

Collaboration calls for long-term commitment and acknowledging that neither change nor collaboration occurs overnight. Civil servants need to be persistent in their involvement in the process, particularly in light of the possibility that contacts might leave again, causing to start the process of collaboration all over again. Furthermore, raising awareness of the importance of a healthy living environment is an ongoing effort. This means that it is important not to avoid discussions when conflicts arise, but to discuss them in a structural manner, keeping in mind the vision of the municipality and expectation management. Taken together, these observations point to the necessity of being committed to the collaborative process, which is continuous.

How do we experience that collaboration, when there is collaboration with the ‘social’ domain? Pff, well. (laughing) Yeah, it was tough, I’ll put it that way. That we’re still apparently so far apart from each other, like… In terms of what we want to achieve. And also with our… The language we speak, maybe, I don’t know. … Yeah, that people … from the ‘social’ domain didn’t want anything at all. We did it twice … We’ve already done that. The first time didn’t turn out to be anything at all. The second time, I did notice that people from the ‘social’ domain had more understanding. They also had more input. Incidentally, they were the same people as the last time. Now we were more open to it than before, for instance. But now, we’re three years on, so, erm*….* (Quotation 6, Municipality E)

The long-term, continuous nature of collaboration raises the issue of time, in combination with tremendous work pressure and lack of capacity. According to participants, this has an enormous influence on collaboration. Investment in the process of collaboration requires making time to (1) come into contact, (2) get to know and understand each other, (3) find common ground between the living environment and health, and (4) get to know the working methods of each domain. Time was also mentioned in combination with the *zeitgeist* (spirit of the times). This refers to the time that it takes for people (from early adapters to late followers) to understand the meaning of collaboration and to develop a solid base for collaboration to jointly achieve the ambitions of the municipality. One positive experience in this regard was observed during the development process of the municipal vision document on environment and planning policy. To date, this process had devoted sufficient attention to time, prioritising themes and organising the vision in a collaborative manner. As mentioned by the participants, from the very beginning, time had been made for coming together, discussing opportunities and finding alignment in perspectives for this vision. In this example, the collaborative process is part of the work process involved in arriving at an overall environment and planning vision. In contrast, within other occasions contacts, the collaborative process is likely to be seen as an additional task added on top other existing tasks. Learning from this positive experience might help to improve collaboration in other occasions.

Another project leader has also arrived, who also has a bit more time and space to think about the process and who has realised, “I shouldn’t do this under a lot of pressure… we should just take time for this.” Which I also think will just give us much more concrete collaborations, including the policy interpretation of a theme like health, but then in a broad sense. (Quotation 7, Municipality A)

Problem definition and conflicts of interest were also identified as having an influence on the collaboration process, although not all participants had experienced the influence of these elements in their own work. Problem definition had influence when issues were approached only from people own perspective or from within a narrow view, thereby ignoring the full picture or allowing for the existence of different perspectives. According to the participants, problem definition can have a particularly heavy influence when a complex problem like health is not seen as an issue, nor the possibility that each domain has an influence on the pursuit of a healthier living environment. Within such contexts, conflicts of interest can arise as well. Aspects mentioned in this regard included the tension between organizational interests and personal interests, the neglect of the ambitions and vision of the municipality, and a focus on short-term as opposed to long-term solutions. Participants noted that there was room for improvement in several areas. One suggestion was to have regular dialog about the implementation of health-related preventive measurements in spatial-planning projects, keeping in mind the long-term effects of these measures. Another was to acknowledge that different people have different points of view, and that they are therefore likely to have different definitions of the problem. Another helpful suggestion could be to start from the same base (e.g., the inhabitants of an area; a mutual assignment). Further suggestions for improvement and details are listed in Table 2.

BOX 1 **What should the social and physical domains do? ideas raised by interviewees**

Participants from the social and physical domains also mentioned improvements for each of the domains.

For the social domain, there is the desire to focus more on doing, coming into action. Participants called for more decisiveness and to become more concrete in this domain. This starts by taking the initiative and communicating where they see linkages with the other domain on various themes. In this process, awareness of the power of repetition and starting by raising awareness and insight can help people in both domains to develop the understanding that they need each other. Another good start could be to invite the physical domain when talking to the neighborhood. The interviewees also noted that the social domain should make projects more a common responsibility at different levels, from start to finish.

For the physical domain, these improvements largely involved being more aware of the social domain and its different disciplines. Making the effort to initiate connections before the start of projects was also mentioned as a specific improvement for the physical domain. Participants also referred to a desire for people in the physical domain to deepen their knowledge of the inhabitants of neighborhoods and start from there. This neighborhood approach is seen as a direct stimulus for collaborating with the social domain, as this domain generally knows more about the people who are living in the municipality. Health should also be a common aspect to incorporate into urban plans by genuinely looking for cross-linkages. Knowing whom to contact and whom to convince in this process at each level is of key importance in this regard. Reserving a budget in this domain for crossing boundaries could also help to enable a more collaborative and integrative approach.

### Institutional design

The institutional design influences collaboration within the organization. Analysis of the data revealed five levels of collaboration that are related to the institutional design and should therefore be considered in the institutional design. First, collaboration takes place at the individual, personal level, between individuals. Second, collaboration occurs between professions and positions, with regard to knowledge and expertise. Third, collaboration between domains takes place to create a more substantive dialog regarding themes, aims and assignments. A fourth level of collaboration is seen between different layers in the municipal organization. Finally, collaboration takes place between the municipal organization and external partners, both public and private. These levels were entangled, thus revealing the layered constitution of collaboration. Given the difference in levels of collaboration, it could be expected that different perspectives also exist concerning how participants understand collaboration as a concept and they experience it in practice.

According to the participants, the institutional design should stimulate collaboration organically. At present, each domain could be seen as a system as such, each with its own policy, processes and issues. This maintains the tendency to think in silos. Rather than drastically changing the organization or requiring the compulsory education of their colleagues, participants suggested changes in the framework of the organization.

… but we’re seeing that now, just based on the instruments we have to make for the Environment and Planning Act — vision and plan — that we’re automatically starting to work together. And that an organization is created automatically. … Very organically, yes. And then we see who we need for that (Quotation 8, Municipality A)

One suggested change was to create teams or overarching programmes (in terms of both people and resources). One example could be a team living environment, which would immediately bring the two worlds together.

If we seek more collaboration, it should be easier to merge budgets. We’re still very much working in boxes, like, “It should all be gone by the end of the year.” Well, my money has run out, because I don’t have that much. (Quotation 9, Municipality C)

Such programmes and teams could also help to overcome the tendency to think in silos, as they work together from the start. This should be done in all layers of the organization, and it should start with clear communicative support and execution at all layers.

One influential element that could stimulate this change comprises culture and language. According to participants, this factor is interwoven throughout the organization, with asymmetries in language (e.g., definitions) and different cultures across domains. One initial step in overcoming this element could be to acknowledge that there are different types of people in each domain, each with a different background and different perspectives on the problem. Take a step away from the problem or issue at the table, and start by searching for similar definitions could help those involved to speak the same language. Participants noted that they should dare to dig deeper and find the root cause and meaning of what someone says until they truly understand what has been said.

Another suggested change had to do with making communication within the organization a more supportive system that could facilitate networking amongst civil servants, easing communicate and linking to each other. Some referred to this as ‘creating a network organization’. In this regard, the organization could also create a sense of unity — a feeling that ‘we’ are working together for the inhabitants. Participants identified this as a facilitating condition, stimulating collaboration that looks over imagined boundaries, even in the heat of the moment and at busy times, as it maintains the focus on the inhabitants. Most participants noted that this condition is not currently present. One structural change that had thus far generated good results in terms of finding each other more easily is to have everyone working in one building. This stimulated small talk through encounters in the corridors or coffee areas. It is also helping to downsize collaboration by allowing civil servants to experience the fact that collaboration starts with small steps.

Sustainable change in the organization can also be created by changing the name of the programme and assigning more in-depth meaning to particular words. This can result in fresh exposure to what is being done. Furthermore, participants suggested that more reflection on the results of policy and collaboration and sharing information about good practices or the process of involvement in a project could create positive experiences and results. This also points to new opportunities for building bridges across domains. If done in a suitable way, such reflection could become a starting position and a sustainable foundation from which to work, as it builds upon previous experience.

To overcome the tendency to think in silos, facilitator jobs could be created within the organization, with bridging the gaps between the social and physical domains included in the job description.

They can indeed bridge that gap. They can think: “Okay, it might be a slightly different language, but I can understand what they mean.” And we can take that on, you know? We need people like that, who have both insight … who are not like either being from ‘social’ or being from ‘physical’, but understanding both. And able to translate it. And also able to translate it within the district. Able to translate to the aldermen. So, we just need them. They are very important. (Quotation 10, Municipality C)

But we also need more and more people who can look outside the box. (Quotation 11, Municipality A)

The creation of such positions was suggested by participants as a way to improve collaboration more organically. In their current experience, participants focus primarily on their daily tasks and the objectives of the own domains, and they continue to be held accountable within this narrow area. Moreover, many participants mentioned that they did not (or did not wish to) have a full picture of what is needed, and that they preferred to focus on their own tasks. The new facilitator should be able to look at the full picture, serve as a linking pin, have knowledge about both worlds (the social and physical domains) and possess expertise with both the content and the process of collaboration.

### Facilitative leadership

Participants expressed a desire for more support. In their opinion, such a supportive system could start with the aldermen and the managers (as mentioned under Starting Conditions). This could generate a structural, directive form of collaboration that emphasises the importance of collaboration.

In the interviews, civil servants also expressed a need for aldermen to dare to consider the long term and frame a clear vision of the municipality. This long-term character should extend beyond the term of the aldermen (in the Netherlands, four years). They should also show their support in both words and actions. This could be encouraged by modeling a joint vision and board assignments, which programme managers can translate to the executive teams. According to the participants, if the board and aldermen were to actively search for ties, the other layers in the organization will follow.

It starts, I think, with shared ambitions in the council, so it’s not just the ambition of one alderman. The whole council should reflect it; they should actually say, "These are our ambitions. Translate them into the programmes.” (Quotation 12, Municipality E)

As mentioned under Institutional Design, facilitators should have leadership skills, in order to bridge the two domains at the executive level and to leverage between levels within the municipal arena. They should also be very straightforward and dare to say, “If you do not have them on board, nothing is going to happen.”

## Discussion

The aim of this study was to unravel municipal civil servants perceptions and experiences with what is needed to enhance collaboration between the social and physical domains within Dutch municipalities, in order to stimulate efforts to build a healthy living environment.

According to the findings, collaboration between the social and physical domains entails a variety of types and levels of collaboration. A crucial factor is apparently a clear understanding of these various levels, the existence of which leads to a variety of interpretations of collaboration. Such an understanding is necessary before improvements can occur. The participants in this study did not seem to be aware of these different levels of collaboration; they simply talked about working together.

The results also indicate that civil servants clearly are willing to work together and that, in recent years, collaboration has improved, as compared to a few years ago. It is also evident that there is a need to work together, although the participants do not yet appear to be fully intrinsically motivated to do this. Such motivation is needed, because collaboration is highly dependent on the individuals involved. The participants also identified several barriers that must be overcome. They suggested concrete improvements to address these barriers.

The improvements suggested above correspond to the various components of the model of collaborative governance (35). Collaboration between the social and physical domains can be strengthened by: (1) having a joint vision and joint assignment, which (2) takes the inhabitants as the starting point; (3) by working in multidisciplinary teams, and (4) by creating jobs to link the domains (boundary spanners). This requires leadership from the level of aldermen and management (34). It also requires an active search for an integrated approach and connecting themes, in addition to emphasising the importance of collaboration between the social and physical domains and being clear in communication (13). In turn, this can create unity (i.e., a sense of togetherness) within the municipality.

The findings clearly indicate that collaboration within municipalities is a continuous learning process, which requires a more open attitude, more mutual exploration and more mutual respect (42). If these crucial aspects are in place, all civil servants could start immediately, seeing collaboration as a crucial part of their work rather than as a supplementary activity.

The model of collaborative governance developed by Ansell and Gash also fits well with the notion that collaboration and aspects for improvement are an ongoing process (35). Although the model helped us to interpret and present the results of this study, our results did not reflect all components that are identified in the model and previous research as being important to collaboration (34, 35). For example, according to the model, the element of trust is a crucial prerequisite for effective collaboration, and it requires immediate attention at the onset of the process. During our interviews, explicit discussion regarding trust was relatively limited. Instead, the participants tended to focus on individuals with whom it may or may not be pleasant to work. Although this might have been due to the trust that these people had in them, we cannot say this that for sure. Regardless, establishing trust was not a topic that received a separate focus at the start of a collaboration, but was a result of the collaboration process over time. In our view, it is more of an intermediate outcome than a starting condition. During the course of collaboration, those involved acquire knowledge on both successful and unsuccessful approaches, in addition to encountering minor successes, and they can make minor adjustments to enhance the collaboration.

The outcomes also do not address the issue of power and disparities in power amongst the participants in a collaboration. This could be because the model assumes collaboration between public organizations and other types of organizations, in which power differences are more obvious. In contrast, we examined collaboration between different domains within a municipality, where the power is organized through a distinct decision-making structure. This nevertheless does not imply that there are no distinct forms of power that can exert influence. For example, having one’s own budget as a policy area or the ability to appoint the project leader could also constitute a form of power that can exert an impact on collaboration and the ultimate outcome thereof. The participants in the interviews apparently did not directly consider the topic of power and who holds it, nor did they consider its implications for collaboration.

In accordance with the concept of power, a mutual dependence is involved in achieving the objective of a healthier living environment (12). It is evident that the integration of public health, which falls under the social domain, depends on the physical domain in order to incorporate health into spatial initiatives. Consequently, the power of the physical domain is more prominent, and the balance of collaboration is not optimal. Such interdependence between the social and physical domains can be made clear by working from a shared vision, which ensures a shared understanding of the problem and a direction for solutions. It marks an important moment at the beginning of the collaboration, and it will be crucial for the remainder of the process. Nonetheless, collaboration amongst civil servants in spatial-planning projects focuses primarily on specific projects, and they are consequently more concerned with executing projects than with developing a joint vision. Collaboration within a project can be more challenging in the absence of a fully agreed-upon vision that guides the implementation of the project, or if the existing vision does not include themes relating to the social domain. Particularly for collaboration within a single organization, having a vision could also be an important starting condition for supporting collaboration.

Supporting the process of collaboration through facilitating leadership is another important component of the model. The findings suggested that municipal collaboration processes currently do not consider this aspect. As indicated by the civil servants participating in the study, it is beneficial to invest in other types of officials (e.g., ‘boundary-spanners’) who could fulfil a bridging function between the social and physical domains (43). The participants spoke primarily about another type of leadership, which must be demonstrated by aldermen and managers. This type of leadership consists of promoting the importance of collaboration, actively establishing connections with diverse disciplines, articulating the fundamental direction of the integrated vision and developing institutional design procedures in a manner that encourages collaboration.

The participants noted that they had experienced problems relating to the manner in which aldermen tend to operate mainly within their own portfolios and in which managers set up processes and organizations structures that hinder collaboration. Our results explicitly point to the responsibility and accountability of these actors in collaboration within municipalities, which has thus far been underexposed. Previous studies have demonstrated that aldermen and managers play an important role in collaboration, and they should ensure that change will happen (35, 44, 45). Direct responsibility was another component of the model that was not explicitly discussed by the participants in this study. This implies that there was a substantial degree of non-binding collaboration between domains. As a result, collaboration continues to depend on intrinsic motivation, which is not stimulated due to various factors, including workload, physical distance and budgeting.

Although Ansell and Gash describe the iterative process in words (35), the visual model of collaborative governance is obviously a simplified representation of how collaboration actually works. In practice, collaboration is an iterative process in which all components of the model play a significant role. Less explicit attention has been paid to the individual components and their respective roles and positions in the collaboration process. Given that the components have many more relationships, interaction and locations in the process of collaboration than the model currently suggests, adding more loops of a centrally positioned aggregation of interactions would be more accurate (46).

### Strength and limitations

The most prominent strength of this study is that it was designed to identify concrete ways in which to enhance collaboration between the social and physical domains and to avoid becoming mired in the obstacles that prevent such collaboration from taking place. The findings of this study clearly reveal a significant level of willingness to establish collaboration. In addition, the results provide many starting points for municipalities. The suggested recommendations might have unintended and undesirable consequences such as the sharing of budgets implies. We recommend that this should be taken into account in the specific municipal context. Because this study is not quantitative the outcomes are not directly applicable to other municipalities. It also does not address disparities amongst municipalities. It would therefore be beneficial to conduct further investigation of similarities and differences through quantitative study in as many municipalities as possible.

Another limitation of this study is that it does not provide insight into the development of collaboration between the social and physical domains. Such insight might have provided interesting starting points for improving collaboration. In addition, the study focused solely on collaboration amongst civil servants from the social and physical domains, and it did not include any other parties involved in spatial-planning projects processes. The roles of aldermen, managers, external parties and inhabitants is also significant in these types of projects, and they can have a significant impact on collaboration and its outcomes.

Future studies could examine the perspectives, contributions and involvement of aldermen, managers, external parties and inhabitants in spatial-planning procedures, as well as the implications of their involvement in the process of collaboration between the social and physical domains. It would also be interesting to focus on specific elements of this collaboration, especially trust and power. Long-term research into the progress of collaboration could generate further knowledge about how such collaboration develops and which elements play more or less prominent role at a certain point. Another interesting area to follow up could concern the possible existence of a relationship of dependence between the physical domain and the social domain. Our findings do not provide a clear picture in this regard. It would therefore be interesting to conduct further investigation to obtain a more precise understanding of this topic.

## Conclusion

The topic of collaboration between the social and physical domain in the Netherlands gains renewed interest and relevance with the introduction of the Environment and Planning Act at the beginning of 2024. The civil servants from Dutch municipalities suggested highly concrete opportunities for improvement within their organizations that could enhance collaboration between the social and physical domains. The participants were aware of their own roles in this regard, and they acknowledged that they could immediately start making contact and initiate the conversation with an open attitude. They also ask aldermen and managers to assume their roles. The primary responsibility of these parties is to communicate the significance of collaboration, highlighting the necessity of executing work with a holistic approach and working on integrating assignments within multidisciplinary teams. The connection between the social and physical domains is naturally present with regard to issues raised by inhabitants, which offer many opportunities for strengthening collaboration. Investments must be made in communication, as well as in people who can focus on establishing a link between the social and physical domains (i.e., ‘boundary spanners’). Improving collaboration can begin immediately if civil servants, managers and administrators regard collaboration as an essential part of their jobs, acknowledge interdependency in achieving their goals and ambitions, and start to develop a shared vision.

## Data availability statement

The datasets presented in this article are not readily available because the qualitative data cannot be anonymized only pseudonymized. The participants of our study can be identified because it concerns a small group of people in specific positions at municipalities in the Netherlands. This makes it possible to find out who said what and when. It is therefore the policy of Radboudumc not to disclose this data in this manner. The data is available on request by Academic Collaborative Centre AMPHI / Radboudumc. Requests to access the datasets should be directed to kwaliteitsteam.elg@radboudumc.nl.

## Ethics statement

The studies involving humans were approved by the medical ethics committee for the Arnhem-Nijmegen area. The studies were conducted in accordance with the local legislation and institutional requirements. The participants provided their written informed consent to participate in this study.

## Author contributions

KM: Conceptualization, Data curation, Formal analysis, Funding acquisition, Investigation, Methodology, Writing – original draft, Writing – review & editing. HS: Conceptualization, Data curation, Formal analysis, Investigation, Methodology, Writing – original draft, Writing – review & editing. KV: Supervision, Writing – review & editing. MB: Supervision, Writing – review & editing. GM: Conceptualization, Supervision, Writing – review & editing.
