# Supplementary material for: Strengthening collaboration within Dutch municipalities for a healthier living environment: experiences and possible improvements according to civil servants
Source: Front Public Health. 2024 Jun 28;12:1406178. doi: 10.3389/fpubh.2024.1406178 (PMC11242545; doi:10.3389/fpubh.2024.1406178)
Supplement: Supplementary file 1 [file Table_1.DOCX]

**Supplementary material**

| **Table Interview guide** | |
| --- | --- |
| Job description | - Can you tell us something about your position? |
| Integrated assessment processes | - How does a spatial planning process work within the municipality? Is there a standard working method/procedure? - How are other policy areas involved and policy documents included in the spatial planning process? - Who is involved or should be involved in the development of a spatial plan? - What do you understand by integrated assessment processes within spatial planning? - How does the municipality make an integrated assessment and decision? - How do you experience the integrated consideration processes surrounding spatial plans within your municipality? - Can you give an example of a project and what decision-making preceded it? - What, in your opinion, are the most important factors when making an integrated assessment? - What changes would you like to see? - How are different interests between policy areas dealt with in a spatial planning process? - What if these interests really conflict? - How is this dealt with and what consideration is made? - What objective criteria are used to make a choice? - Do you have the idea that one policy field has more priority than the other in integrated considerations in spatial planning? What factors do you think are responsible for this? What is your opinion about this? How would you like to see this differently? |
| Collaboration social and physical domain | - What do you understand by the physical domain and what do you understand by the social domain? - How is there collaboration between the social and physical domains within your municipality? - What is your relationship like with people from other departments? - Who do you have the most contact with? (people from the social and/or physical domain) - How do you experience the collaboration between the social and physical domains within your municipality? - Do you think the current level of collaboration is sufficient? If yes or no, what is the reason? - What issues hinder this collaboration? - What measures do you think could be taken to promote this collaboration? - Various obstacles have already emerged from previous research. Would you like to put these in order of perceived obstacles in your own organization?   Obstacles: Different problem definition, Conflicting interests, Difference in vision (short/long term), Cultural and linguistic differences, Lack of mutual understanding |
| Health in spatial planning projects | - How is health taken into account in the spatial planning process within your municipality? - This is an overview of various stages in which attention is paid to health in spatial plan development. Can you indicate which phase you think your municipality is in? (Overview of stages)   Five stages: unrecognised, recognised, considered, implemented, integrated and institutionalised   - What are the limiting factors in including health in spatial planning? - What are factors that promote the inclusion of health in spatial planning? - What could your health or social domain policy advisor do to include health in spatial planning plans? - What could the physical domain do to include health more in spatial planning plans? |
| Environment and Planning Act | - How does your municipality anticipate the introduction of this law? - Have “pilot projects” already been set up that (partly) work as if the law had already been introduced? - To what extent do you think the law will influence the policy of your municipality? - What expectations do you have of the introduction of the Environment and Planning Act? - What are the most positive points? - What do you have your doubts about? - How do you think that the introduction of the Environment and Planning Act will create more room for health within spatial planning? |
| General information | - What education did you received? - What is the official title of your position within the municipality? - How long have you held this position? - Did you previously hold another position within the municipality? If yes, which one? Why the change in position? |
